# Condensation of the RNA chaperone Hfq is coupled to inhibition of glucose uptake and contributes to the stabilization of regulatory RNAs in nitrogen-starved *Escherichia coli*

**DOI:** 10.1093/nar/gkaf1006

**Published:** 2025-10-21

**Authors:** Josh McQuail, Harriet R Ellis, Volker Behrends, Cristina Balcells, Thorsten Bischler, Tom Gräfenhan, Sivaramesh Wigneshweraraj

**Affiliations:** Department of Infectious Disease, Section of Molecular Microbiology and Centre for Bacterial Resistance Biology, Imperial College London, London SW7 2AZ, United Kingdom; Department of Infectious Disease, Section of Molecular Microbiology and Centre for Bacterial Resistance Biology, Imperial College London, London SW7 2AZ, United Kingdom; School of Medicine and Biosciences, University of West London, London W5 5RF, United Kingdom; Department of Surgery and Cancer, Institute of Reproductive and Developmental Biology, Imperial College London, London W12 0NN, United Kingdom; Department of Surgery and Cancer, Institute of Reproductive and Developmental Biology, Imperial College London, London W12 0NN, United Kingdom; Core Unit Systems Medicine, University of Würzburg, Würzburg D-97080, Germany; Core Unit Systems Medicine, University of Würzburg, Würzburg D-97080, Germany; Department of Infectious Disease, Section of Molecular Microbiology and Centre for Bacterial Resistance Biology, Imperial College London, London SW7 2AZ, United Kingdom

## Abstract

Ribonucleoprotein condensates are membraneless compartments that concentrate RNA-binding proteins and RNA, and play key roles in cellular adaptation across both eukaryotes and bacteria. While the biological roles of ribonucleoprotein condensates are better understood in eukaryotic systems, the knowledge of metabolic processes that govern their formation and their contribution to stress adaptation remains at a nascent stage in bacterial RNA biology. Hfq is an RNA chaperone conserved in many bacteria that undergoes condensation in response to diverse stresses. Using nitrogen (N) starvation in *Escherichia coli* as a model stress condition, we show that Hfq condensation occurs independently of any extracellular metabolic cues, cytoplasmic shrinkage that cells undergo during N starvation, or the canonical NtrBC-dependent adaptive response to N starvation. We demonstrate that Hfq condensation is coupled to α-ketoglutarate-dependent inhibition of glucose uptake in N-starved *E. coli*. Further, by comparing the transcriptomes of wild-type bacteria and bacteria unable to form Hfq-condensates, we reveal that Hfq-condensates contribute to the maintenance of Hfq-associated non-coding regulatory RNAs during N starvation. We propose that coordination of carbon and N metabolism during N starvation, critical for metabolic adaptation, is accompanied by preservation of non-coding regulatory RNAs via Hfq condensation.

## Graphical Abstract

**Graphical Abstract:**
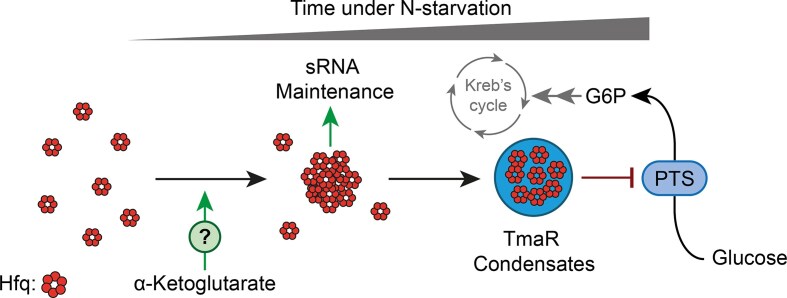

## Introduction

Bacteria often exist in a growth-attenuated state for long periods of time, as many environments inhabited by bacteria are limited for nutrients. As such, bacteria exhibit a range of adaptive responses to long-term nutrient starvation. Many of these are underpinned by changes in gene expression at the level of RNA metabolism. At the level of RNA synthesis, specific transcription regulators respond to cellular cues and modulate the activity of the RNA polymerase, reprogramming the transcriptional landscape and thereby activating or downregulating specific metabolic pathways. In parallel, regulatory non-coding RNA molecules, hereafter referred to as small RNAs (sRNA), mediate the post-transcriptional regulation of gene expression in bacteria. Some sRNAs are *cis*-acting and are expressed from the same locus as their sole messenger RNA (mRNA) target. However, the majority of sRNAs are *trans*-acting and target multiple mRNA targets at different genetic loci. As such, unlike *cis*-acting sRNAs, *trans*-acting sRNAs share only partial complementarity with their mRNA targets and significantly depend on RNA-binding chaperone proteins to interact with their targets. sRNA can affect the fate of their cognate mRNA either positively or negatively in several ways (reviewed in [1–3]).

Nitrogen (N) is used for the biosynthesis of the building blocks of all proteins (amino acids), nucleic acids (nucleotides), and numerous metabolites and cofactors in bacteria. As such, N is an essential component of the bacterial cell. Many groups have used *Escherichia coli* as the model bacterium to understand the adaptive response to N-starvation; thus, the transcriptional regulatory basis of the adaptive response to N-starvation is well understood [4]. Briefly, this involves the two-component system, NtrBC, in which the phosphorylation of the response regulator NtrC by its cognate sensor kinase NtrB allows NtrC-P to activate the transcription of ∼100 genes. These include *relA*, the gene responsible for the synthesis of the RNA polymerase binding stress signalling nucleotide guanosine pentaphosphate [5], and *nac*, which encodes a global transcription regulator & nitrogen assimilation control protein, Nac [6]. Consequently, this leads to a large-scale reprogramming of the transcriptome to allow N scavenging from alternative sources of N, concomitant with the quiescing of cellular processes that consume N [5, 7, 8]. In contrast, very little is known about the post-transcriptional regulatory basis of the adaptive response to N starvation in bacteria. Emerging results have now begun to shed light on the role of Hfq-dependent sRNAs in N-starved *E. coli*. Walling *et al.* showed that the sRNA GlnZ promotes cell survival by regulating genes linked to nitrogen and carbon flux in N-starved *E. coli* (see later) [9]. McQuail *et al.* used genome-scale analyses to reveal that Hfq-mediated sRNA–mRNA interactions in *E. coli* are extensive and dynamic during N starvation [10], indicating a continuous requirement for post-transcriptional regulation during N starvation.

We discovered that ∼50% of all Hfq molecules gradually assemble into foci-like subcellular structures near the cell poles as a function of time under N starvation [11, 12]. Henceforth, we refer to these foci of Hfq as ‘condensates’ as they form by a process analogous to liquid–liquid phase separation [11] and are reversible, i.e. they disperse when N is replenished [11, 12]. In a subsequent study, Goldberg *et al.* showed that Hfq-condensates also exist in stationary phase *E. coli* grown in lysogeny broth or in bacteria experiencing osmotic shock, suggesting that the Hfq-condensates are a prevalent feature in the subcellular landscape of stressed bacteria [13]. The Hfq-condensates that form in N-starved *E. coli* co-localize with condensates of the RNA-degradosome [11] but this was not the case for Hfq condensates that form in stationary phase and osmotically stressed *E. coli* [13]. However, the Hfq condensates that form in stationary phase or osmotically stressed bacteria co-localize with a condensate formed by a protein called TmaR [13]. The TmaR protein binds to and inhibits the phosphotransferase system (PTS) enzyme I, and the presence of TmaR condensates is indicative of inhibition of sugar uptake [14, 15]. Notably, while the formation of Hfq condensates depends on TmaR condensates in stationary phase and osmotically stressed bacteria, the formation of TmaR condensates does not depend on Hfq condensates. Although it is not known whether Hfq condensation depends on TmaR condensates under N starvation, the interplay between Hfq condensation, TmaR, and regulation of sugar uptake is not fully understood for any stress condition under which Hfq condensates have been observed.

We showed that the efficacy by which the Hfq condensates form in N-starved *E. coli* is affected by the RNA binding activity of Hfq [12]. Consistent with this view, Goldberg *et al.* demonstrated that RNA is a constituent of the Hfq-condensates by assembling Hfq-condensates *in vitro*. This study further showed that some polar-enriched RNA molecules in osmotically stressed bacteria, including sRNAs, were destabilized in Δ*tmaR* bacteria (which do not contain Hfq-condensates) [13, 16]. A recent study by Guan *et al.* [17] proposed that Hfq-condensates serve as a nucleoprotein hub to protect mRNA transcripts while simultaneously facilitating the degradation of transcripts less essential for survival and growth resumption. Given that one of Hfq’s canonical functions is sRNA stabilization, it is possible that Hfq condensates also serve as a hub for sRNA stabilization during stress, but this remains to be experimentally demonstrated.

Given that Hfq condensates represent a recent discovery in bacterial post-transcriptional regulation, the cellular mechanisms underlying their formation and their role in stress adaptation remain incompletely understood. Here, we allowed *E. coli* cells to become N starved by growing them in a defined medium containing limiting ammonium chloride (sole N source) and excess glucose (sole carbon source). We then investigated the factors that drive Hfq condensation, delineate the interplay between Hfq condensates, TmaR, and inhibition of glucose uptake, and establish a functional role for Hfq condensates in the adaptive response to N starvation in *E. coli*

## Materials and methods

### Bacterial strains and plasmids

All strains used in this study were derived from *E. coli* K-12 and are listed in Supplementary Table 1. The TmaR-PAmCherry strain was constructed using the λ Red recombination method [18] to create an in-frame fusion encoding a linker sequence and PAmCherry, followed by a kanamycin resistance cassette (amplified from the Hfq-PAmCherry strain) to the 3′ end of *tmaR*. Gene deletions (Δ*tmaR*, Δ*ppK*, Δ*glnB*,or Δ*glnZ*) were introduced into the WT or Hfq-PAmCherry strains as described previously [5]: briefly, the knockout alleles were transduced using the P1*vir* bacteriophage with strains from either the Keio collection [19] or provided by the Storz lab (Δ*glnZ* [9]), serving as donors.

### Bacterial growth conditions

N starvation experiments were conducted as described in [8]. Briefly, unless otherwise stated, bacteria were grown in Gutnick minimal medium (33.8 mM KH_2_PO_4_, 77.5 mM K_2_HPO_4_, 5.74 mM K_2_SO_4_, 0.41 mM MgSO_4_), supplemented with Ho-LE trace elements [20], and 0.4% (w/v) glucose, and 10 mM NH_4_Cl (for overnight cultures) or 3 mM NH_4_Cl (for N starvation experiments) at 37°C in a shaking (180 rpm) incubator. For experiments involving spent media: cultures of WT MG1655 *E. coli* were first grown to N-24 and centrifuged, the resulting ‘spent’ media was then sterile filtered before use. Bacteria from either N+ (N replete conditions) or N− (upon N run-out in growth medium) were centrifuged, resuspended in spent media, and incubated at 37°C in a shaking (180 rpm) incubator. For experiments involving dimethyl-ketoglutarate (dmKG), dmKG was added directly into the culture to a final concentration of 40 mM at N−. The proportion of viable cells in the bacterial population was determined by measuring CFU/ml from serial dilutions on lysogeny broth agar plates.

### Photoactivated localization microscopy and single-molecule tracking

For the photoactivated localization microscopy (PALM) and single-molecule tracking (SMT) experiments, the Hfq-PAmCherry (and derivatives) and TmaR-PAmCherry reporter strains were used. Bacterial cultures were grown as described earlier, and samples were taken at the indicated time points, then imaged and analysed as previously described [21, 22]. Briefly, 1 ml of culture was centrifuged, washed, and resuspended in a small amount of Gutnick minimal medium without any NH_4_Cl + 0.4% glucose; samples taken at N+ were resuspended in Gutnick minimal medium with 3 mM NH_4_Cl + 0.4% glucose; samples in C-source experiments were resuspended in Gutnick minimal media without either NH_4_Cl or glucose. One μl of the resuspended culture was then placed on a Gutnick minimal medium agarose pad [1× Gutnick minimal medium with no NH_4_Cl + 0.4% glucose with 1% (w/v) agarose]; samples taken at N+ were placed on a pad made with Gutnick minimal medium with 3 mM NH_4_Cl, samples in C-source experiments were placed on a pad made with Gutnick minimal media without either NH_4_Cl or glucose. Cells were imaged on a PALM-optimized Nanoimager (Oxford Nanoimaging, https://oni.bio/nanoimager/) with 15 ms exposures, at 66 frames per second over 10 000 frames. Photoactivatable molecules were activated using 405 and 561 nm lasers. Fields of view typically consisted of 100–200 bacterial cells. For SMT, the Nanoimager software was used to localize the molecules by fitting detectable spots of high photon intensity to a Gaussian function. The Nanoimager software SMT function was then used to track individual molecules and draw trajectories of individual molecules over multiple frames, using a maximum step distance between frames of 0.6 μm and a nearest-neighbour exclusion radius of 0.9 μm. The software then calculated the apparent diffusion coefficients (D*) for each trajectory over at least four steps, based on the mean squared displacement of the molecule. To calculate %H_IM_, we collated D* values from multiple fields of view and determined the proportion of D* values that fell into our previously defined immobile population (D* ≤0.08 μm/s^2^). To calculate the proportion of cells with condensates, PALM datasets were first analysed using the cluster analysis function of CODI (Oxford Nanoimaging, https://alto.codi.bio/). Both Hfq and TmaR condensates were analysed using DBSCAN*: Hfq condensates were defined with an eps distance of 75 nm and filtered to those with >50 localizations/cluster and a density >0.0015 localizations/nm^2^, TmaR condensates were defined with an eps distance of 100 nm and filtered to those with >100 localizations/cluster. Total number of condensates per field of view was then used to determine the portion of total cells that contained condensates.

### Immunoblotting

Immunoblotting was conducted in accordance with standard laboratory protocols, with primary antibodies incubated overnight at 4°C. The following antibodies were used: rabbit polyclonal anti-mCherry (abcam, ab167453), mouse monoclonal anti-DnaK antibody (Enzo, 8E2/2) at 1:1000 dilution, HRP goat anti-mouse IgG (BioLegend, 405 306) at 1 : 10 000 dilution and HRP goat anti-rabbit IgG (GE Healthcare, NA934) at 1:10 000 dilution. ECL Prime western blotting detection reagent (GE Healthcare, RPN2232) was used to develop the blots, which were analysed on the ChemiDoc MP imaging system, and bands quantified using Image Lab software.

### Glucose assay

Glucose concentration in the media was determined using the Glucose Assay Kit (Sigma–Aldrich, MAK476) in accordance with the manufacturer’s protocol.

### Targeted metabolite measurement

At the indicated time points, ∼10^10^ cells were collected, and washed twice in ¼ strength Ringer’s solution (Thermo Scientific, BR0052G). Cell pellets were resuspended in 500 μl of cold (−20°C) methanol:acetonitrile:water (2:2:1, v/v/v) + 0.1% formic acid (FA). Samples were stored at −80°C until analysis. Before analysis, all vials received were reconstituted with 150 μl of 97.5% H_2_O + 2.5% acetonitrile + 0.2% FA, diluted, vortexed, and transferred to inserts. Pooled quality control of all samples was then generated by pooling 10 μl of the first replicate for each experimental condition and injected every eight samples. All reagents used were of ultra-high-performance liquid chromatography gradient grade, and all standards were of analytical grade. Targeted metabolomics analysis was performed using an Agilent 1290 liquid chromatography (LC) system (Agilent Technologies, CA, USA) coupled to a QTRAP 4000 mass spectrometry (MS) system (SCIEX, Danaher, WA, USA). Chromatographic separation was achieved using a Luna Omega Polar C18 column (Phenomenex/Danaher, WA, USA). The analysis was conducted in positive ion mode (A: H2O + 0.2% FA / B: acetonitrile + 0.2% FA) on a 20-min gradient and in negative ion mode (A: H_2_O + 0.1% FA + 10 mM ammonium formate / B: 100% acetonitrile) on a 14-min gradient at 0.450 ml/min flow rate. All data was acquired in multiple reaction monitoring mode.

Resulting spectra analysed using an in-house data analysis workflow based on [23].

### T7 phage infection assay

Bacterial cultures were grown in Gutnick minimal medium as described above to the indicated time points. Bacterial culture samples were taken, centrifuged and resuspended in fresh Gutnick minimal media supplemented with either 2 mM NH_4_Cl and 12.5 mM glucose (for N+) or 5 mM glucose (for N-24, i.e. 24 h following N-) and diluted to *A*_600 nm_ of 0.3 to a final volume of 500 μl and transferred to a flat-bottomed 48-well plate, together with T7 phage at a final concentration of 4.2 × 10^9^ phage/ml. The cultures were then grown at 37°C with shaking at 700 rpm in a SPECTROstar Nano microplate reader (BMG LABTECH), and *A*_600 nm_ readings were taken every 10 min.

### RNA sequencing

WT and Δ*tmaR* bacteria were harvested at N-3 (i.e. 3 h following N-) and N-24; additionally, cultures from N-24 were treated with 100 μg/ml rifampicin, with further samples taken following 15- and 60-min of incubation. N-24 and *T* = 0 refer to the same samples and extracted/sequenced RNA. RNA was extracted using the RNAsnap protocol [24]. Three biological replicates of each strain were taken and mixed with a phenol:ethanol (1:19) solution at a ratio of 9:1 (culture:solution) before harvesting the bacteria immediately by centrifugation. Pellets were resuspended in RNA extraction solution (18 mM ethylenediaminetetraacetic acid, 0.025% sodium dodecyl sulphate, 1% 2-mercaptoethanol, 95% formamide) and lysed at 95°C for 10 min. Cell debris was pelleted by centrifugation. RNA was purified with PureLink RNA Mini Kit extraction columns (Invitrogen, 12183018A) and largely in accordance with the manufacturer's protocol for Total Transcriptome Isolation except with a final ethanol concentration of 66% to increase the yield of smaller RNA species. Analysis of extracted RNA was performed following depletion of ribosomal RNA molecules using a commercial rRNA depletion kit for mixed bacterial samples (Lexogen, RiboCop META, #125). The ribo-depleted RNA samples were first fragmented using ultrasound (4 pulses of 30 s at 4°C). Then, an oligonucleotide adapter was ligated to the 3′ end of the RNA molecules. First-strand cDNA synthesis was performed using M-MLV reverse transcriptase with the 3′ adapter as primer. After purification, the 5′ Illumina TruSeq sequencing adapter was ligated to the 3′ end of the antisense cDNA. The resulting cDNA was PCR-amplified using a high-fidelity DNA polymerase and the barcoded TruSeq-libraries were pooled in approximately equimolar amounts. Sequencing of pooled libraries, spiked with PhiX control library, was performed at a minimum of 10 million reads per sample in single-ended mode with 100 cycles on the NextSeq 2000 platform (Illumina). Demultiplexed FASTQ files were generated with bcl-convert v4.3.6 (Illumina). Raw sequencing reads were subjected to quality and adapter trimming via Cutadapt [25] v2.5 using a cutoff Phred score of 20 and discarding reads without any remaining bases (parameters: –nextseq-trim = 20 -m 1 -a AGATCGGAAGAGCACACGTCTGAACTCCAGTCAC). Afterwards, all reads longer than 11 nt were aligned to the *E. coli* K12 MG1655 reference genome (Genbank assembly accession: GCA_000005845.2) using the pipeline READemption [26] v2.0.3 with segemehl version 0.3.4 [27] and an accuracy cut-off of 95% (parameters: -l 12 -a 95). READemption gene_quanti was applied to quantify aligned reads overlapping genomic features by at least 10 nt (-o 10) on the sense strand based on GenBank annotations (CDS, ncRNA, rRNA, tRNA) for assembly GCA_000005845.2 from Dec 7, 2024. Based on these counts, differential expression analysis was conducted for the N-3 and N-24 transcriptomes via DESeq2 [28] version 1.24.0. Read counts were normalized by DESeq2 and fold-change shrinkage was conducted by setting the parameter betaPrior to TRUE. Differential expression was assumed at adjusted *P*-value after Benjamini-Hochberg correction (padj) < 0.05 and |log2FoldChange| ≥ 1. Results of the DESeq2 analysis can be found in Supplementary Data 1. For the rifampicin-treated samples (RIF-seq), read counts were normalized to the reads of 6S RNA (*ssrS)* from the respective sample. Results for the RNA turnover analysis can be found in Supplementary Data 2.

## Results

### Hfq condensation is a response exclusive to intracellular metabolic changes

**Figure 1.**
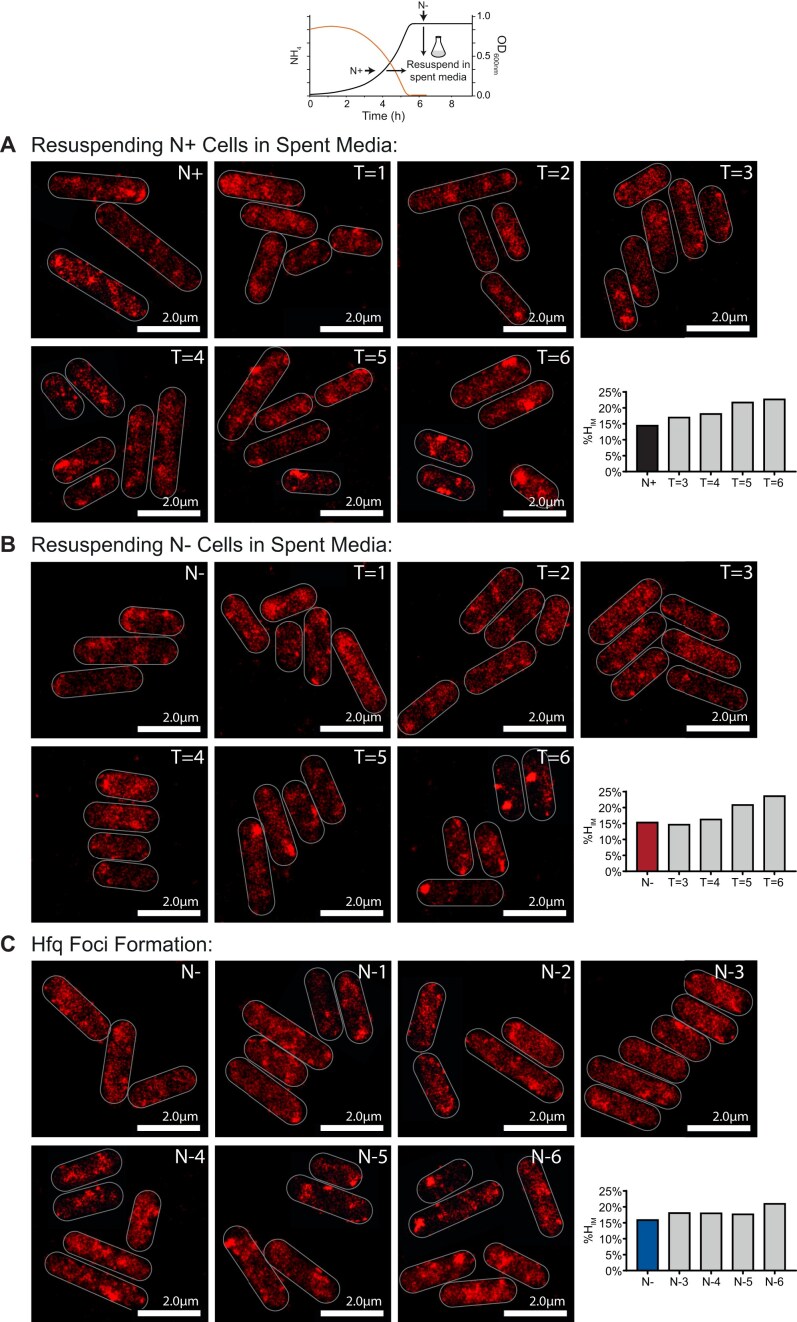
Hfq condensation is a response exclusively to intracellular metabolic changes. Representative PALM images of Hfq in *E. coli* after cells from (**A**) N+ or (**B**) N- were resuspended in spent media from N-24 cultures and imaged hourly. (**C**) *E. coli* cells experiencing N starvation for 6 h, imaged hourly. Graphs show %H_IM_ values.

Our experimental system involves growing a batch culture of *E. coli* strain MG1655 (which contains photoactivatable mCherry tag fused to the C-terminal end of *hfq*) in a defined minimal growth medium in the presence of a limiting amount (3mM) of ammonium chloride as the sole N source and excess (22mM) glucose as the carbon (C) source [12]. Under these conditions, when ammonium chloride in the growth medium runs out (N-), the bacteria enter a state of N starvation and become growth-arrested. However, sufficient glucose still remains to support growth if the N source becomes replenished [12]. The Hfq-condensates are not detected at N- but form gradually as N starvation ensues and become detectable ∼6 h into N starvation (N-6) and, by 24 h following N-starvation (N-24) ∼80%–90% of all cells in the population contain the Hfq-condensates. Further, Hfq-condensates also form in bacteria that have been treated (at N-) with rifampicin (transcription inhibitor) or chloramphenicol (translation inhibitor), implying that Hfq-condensates do not depend on *de novo* gene expression during N starvation [12]. We considered whether Hfq condensation is triggered in response to an extracellular cue, i.e. a signalling metabolite, that accumulates over time under N starvation. To investigate this, we used bacteria from N+ (when cells are not N-starved, and Hfq-condensates are absent) and N- (when cells are N-starved, but Hfq-condensates are absent) and resuspended the bacteria in ‘spent’ media from cultures of bacteria grown to N-24. Although the spent media contains glucose (see above; [12]), it does not have any N source to support growth. We then periodically monitored the diffusion dynamics of individual Hfq molecules by PALM in live cells. We reasoned that if an extracellular cue existed, then Hfq-condensates formation would occur faster in spent media (in which such a cue would have accumulated) than it would when bacteria become progressively more N-starved over time in normal (unspent) media. A quantitative parameter to measure Hfq-condensate formation, %H_IM_, was calculated as the proportion of total Hfq molecules with an apparent diffusion <0.08 μm^2^/s, which had previously been defined as the ‘immobile’ population of Hfq molecules [12]. The %H_IM_ was calculated based on all trajectories of Hfq in 50–200 bacterial cells within a given field of view. We did not detect any differences in %H_IM_ as a function of time following inoculation of bacteria from N+ (Fig. 1A) or N- (Fig. 1B) into spent media and, in both cases, the Hfq-condensates became detectable and clearly distinguishable from the mobile population of Hfq molecules ∼6 h following incubation; the rate of Hfq condensation in both cases was similar to that observed in bacteria becoming progressively N-starved in unspent media (Fig. 1C). We conclude that Hfq condensation is not mediated by any extracellular cues but represents a response exclusive to metabolic changes that occur inside *E. coli* cells during N-starvation.

### Cytoplasmic shrinkage during N starvation does not contribute to Hfq condensation

**Figure 2.**
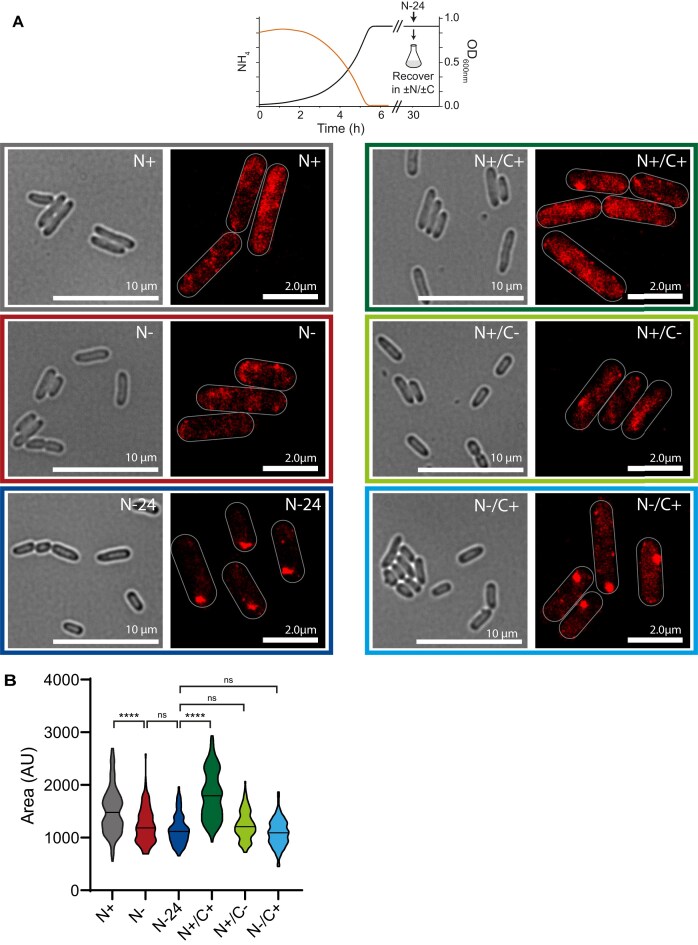
Cytoplasmic shrinkage during N starvation does not contribute to Hfq condensation. (**A**) Representative phase contrast and PALM images of Hfq in *E. coli* at different stages of N starvation (N+, N−, and N-24) and 2 h after N-24 cells were resuspended in fresh media with different combinations of N and C (N+/C+, N+/C− and N−/C+). (**B**) Violin plot of cytoplasmic area determined at each measured time point; the median area is indicated with the horizontal line. Statistical analysis performed by Kruskal-Wallis test with Dunn's multiple comparisons. (****P< 0.0001).

It is well established that the cytoplasm of *E. coli* cells shrinks during nutrient starvation [29, 30]. This inevitably increases the density of the cytoplasm, inducing macromolecular crowding and thereby affecting the diffusibility of macromolecules. We thus considered whether progressive shrinkage of the cytoplasm in response to N-starvation could trigger Hfq condensation. Therefore, we measured the average cytoplasmic area of bacterial cells and correlated this with the presence and dispersion of the Hfq-condensates. As shown in Fig. 2, as expected, the average cytoplasmic area decreased from ∼1509 arbitrary units to ∼1235 arbitrary units when N+ bacteria became N-starved. Notably, no difference in the average cytoplasmic area was detected between N- (Hfq-condensates absent) and N-24 (Hfq-condensates present) bacteria. When N-24 bacteria, where the Hfq-condensates have fully formed, were resuspended in fresh media that supported growth recovery (ammonium chloride and glucose present; N+/C+) and led to the dispersion of the Hfq-condensates, the average cytoplasmic area reverted to that seen in N+ bacteria. However, when N-24 bacteria were resuspended in fresh media which only contained ammonium chloride but no glucose (N+/C-) and hence did not support growth recovery but still caused the dispersion of the Hfq-condensates, the average cytoplasmic area did not increase and resembled that seen in N-24 bacteria (where the Hfq-condensates exist). Conversely, in control experiments when N-24 bacteria were resuspended in fresh media that contained no ammonium chloride but only glucose, where the Hfq-condensates *did not* disperse, the average cytoplasmic area also did not increase and resembled that seen in N-24 bacteria. Overall, we conclude that cytoplasmic shrinkage during N starvation does not contribute to Hfq condensation.

### Hfq condensation in N-starved *E. coli* requires TmaR

**Figure 3.**
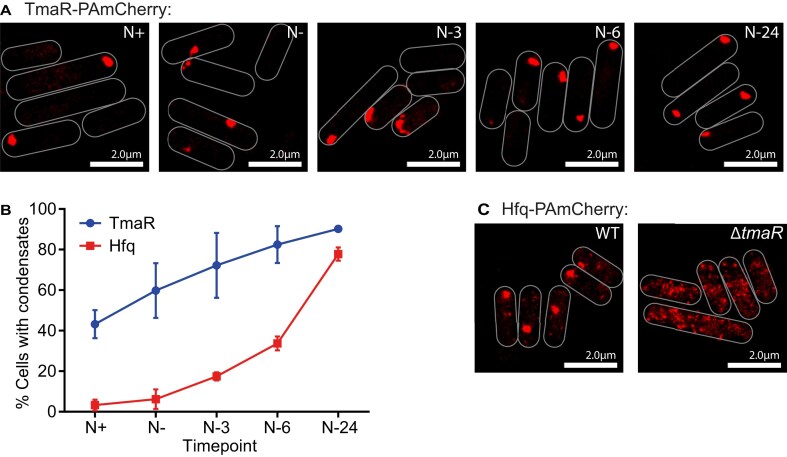
Hfq condensation in N-starved *E. coli* requires TmaR. (**A**) Representative PALM images of TmaR in *E. coli* as a function of time under N starvation. Images were taken at the indicated time points. (**B**) Graph showing the proportion of cells with detectable TmaR (blue) or Hfq (red) condensates during N starvation. Error bars represent standard deviation (*n* > 3). Larger fields of view of the data represented in panels (A) and (B) can be found in Supplementary Fig. 1. (**C**) Representative PALM images of Hfq in WT and Δ*tmaR* bacteria at N-24.

Based on our results thus far, we focused our investigation on intracellular events that drive or require Hfq condensation. The dependency of Hfq-condensates on TmaR has been demonstrated in late stationary phase and osmotically stressed *E. coli* but not in N-starved *E. coli*. In both stress conditions, the Hfq-condensates and the TmaR-condensates colocalize [13]. To investigate the role of TmaR on Hfq condensation under N-starvation, we compared the diffusion dynamics of Hfq and TmaR (containing a photoactivatable mCherry tag fused to its C-terminal end) by PALM. The percentage of cells containing the TmaR-condensates steadily increased during N starvation and, by N-24 ∼80% of cells contained the TmaR-condensates (Fig. 3A and B; Supplementary Fig. 1). In N-24 Δ*tmaR* bacteria we did not detect any Hfq-condensates (Fig. 3C). We note that, unlike the Hfq-condensates, under our conditions, a substantial proportion of cells contain TmaR-condensates before the Hfq-condensates become detectable. For example, at N+ and N−, when Hfq-condensates are entirely absent, ∼40% and ∼60% of the cells contained TmaR-condensates. We conclude that although Hfq condensation strictly depends on TmaR-condensates, Hfq-condensates do not form concomitantly with TmaR-condensates but form subsequently to them. Further, it is known that glucose uptake rates in N-starved *E. coli* decreases more drastically than in any other stress condition [31]. As the TmaR condensation leads to the inhibition of PTS enzyme I [14, 15], the results imply that the inhibition of glucose uptake in bacteria entering N starvation is not homogenous.

### Exogenous addition of α-ketoglutarate induces Hfq condensation

**Figure 4.**
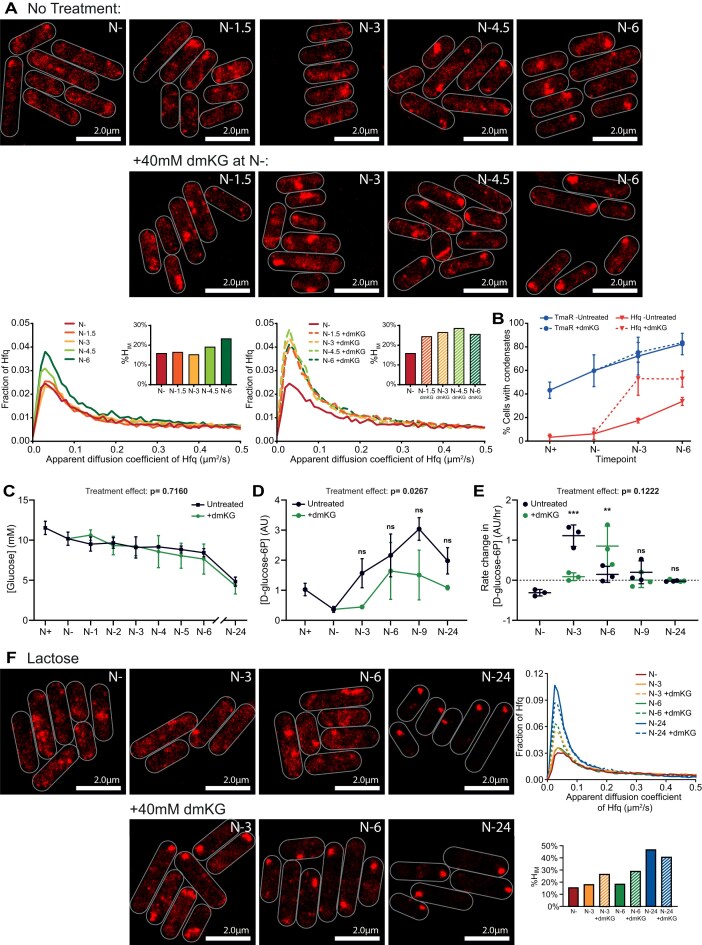
Hfq condensation in N-starved *E. coli* is coupled to inhibition of glucose uptake. (**A**) Representative PALM images of Hfq in *E. coli* experiencing N starvation with and without treatment with 40 mM dmKG at N-. Graphs show the distribution of apparent diffusion coefficients of Hfq molecules at the different sampling time points and the corresponding %H_IM_. (**B**) Graph showing the proportion of cells with detectable TmaR (blue) or Hfq (red) condensates during N starvation; the dashed line indicates the proportion of cells with detectable condensates after adding dmKG at N-. Errors bars represent standard deviation (*n* > 3). (**C**) Graph showing the concentration of glucose in the media of cultures of *E. coli* experiencing N starvation with (green) and without (black) treatment with 40 mM dmKG at N-. Errors bars represent standard deviation (*n* = 3). (**D**) As in panel (C) but of intracellular concentration of D-glucose-6-phosphate (G6P). (**E**) Graph showing the rate change in G6P concentration; values are shown as a proportion relative to the previous time point divided by time between time points (*n* = 3). (**F**) as in panel (A), but for bacteria grown in media with lactose as the sole C-source. Larger fields of view of the data presented in panels (A) and (F) can be found in Supplementary Fig. 2. Statistical analysis performed by two-way ANOVA with Šidák multiple comparisons. (***P< 0.001; *P< 0.05).

Nitrogen starvation reduces the flux of α-ketoglutarate (αKG) out of the Krebs cycle into amino acids biosynthesis, leading to a rise in αKG levels, repression of glucose uptake and inhibition of cAMP synthesis. The latter prevents the global transcription factor CRP from activating transcription of genes required for uptake and catabolism of other sugars. Thus, αKG is a critical metabolite for coordinating the regulation of sugar uptake during N starvation in *E. coli*. During growth in glucose, a particularly critical interaction linking N availability to glucose uptake is the inhibition of the PTS enzyme I, the target of TmaR [14, 15], by αKG [32]. As Hfq condensation depends on TmaR condensation, we speculated whether addition of exogenous αKG to N- bacteria, when Hfq-condensates are absent would induce Hfq condensation (recall that Hfq-condensates only become detectable by N-6). As shown in Fig. 4A and B and Supplementary Fig. 2, when dmKG, a membrane-permeable ester that is cleaved by intracellular esterases to form αKG, was added to N- bacteria, Hfq condensation could be induced and Hfq-condensates formed much sooner in dmKG treated compared to untreated bacteria. Control experiments showed that the dynamics of the TmaR-condensates remained unaffected by dmKG (Fig. 4A and Supplementary Fig. 3A) and that dmKG induction of Hfq-condensates was dependent on TmaR (Supplementary Fig. 3B). Guan *et al.* [17] revealed that long-chain polyphosphates (polyP), which accumulate during N starvation, serve as a scaffold for the assembly of the Hfq-condensates. Additional control experiments using Δ*ppK* bacteria showed that dmKG induction of Hfq-condensates was also dependent on polyP (Supplementary Fig. 3C). Further, no detectable difference in Hfq levels was observed between dmKG untreated and treated bacteria (Supplementary Fig. 3D). Notably, Hfq-condensates could be induced with dmKG even in the presence of the transcription inhibitor rifampicin and translation inhibitor chloramphenicol, suggesting that the induction of Hfq condensation by dmKG occurs independently of any changes in gene expression due to the addition of dmKG (Supplementary Fig. 4). The adaptive response to N starvation requires the two-component system NtrBC, where NtrB is the sensor kinase and NtrC the transcription regulator (see Introduction). At a low αKG concentration (e.g. under N-replete conditions), GlnB (PII) binds to αKG, which allows it to interact with NtrB, inhibiting its kinase activity and activating its phosphatase activity to dephosphorylate NtrC, rendering the latter unable to activate transcription. However, at higher αKG concentrations (e.g. upon N starvation), GlnB binds additional molecules of αKG and is unable to interact with NtrB, such that NtrB acts as a kinase to phosphorylate NtrC, allowing it to activate transcription of genes required for adaptation to N starvation (Supplementary Fig. 5A). We thus considered whether dmKG induces Hfq condensation by overstimulating the NtrC regulon by binding to GlnB. However, Hfq-condensates could still be induced with dmKG in Δ*glnB* bacteria (Supplementary Fig. 5B and C). Therefore, it seems that, during N starvation, the condensation of Hfq is a response that occurs independently of the canonical NtrBC-dependent adaptive response pathway. This view is further supported by the fact that Hfq condensation, in both dmKG untreated [12] and treated (Supplementary Fig. 4) bacteria occurs independently of *de novo* gene expression, consistent with our previous observation that Hfq condensates are present in bacteria devoid of NtrC [12].

### The inhibition of the PTS system in N-starved *E. coli* happens concomitantly with Hfq condensation

Given that the addition of dmKG induces the Hfq-condensates (Fig. 4A and B), that αKG contributes to the inhibition of glucose uptake via inhibiting PTS enzyme I (see earlier) and that Hfq-condensates can form independently of the NtrBC pathway (Supplementary Fig. 5B and C), we considered whether Hfq condensation in N-starved *E. coli* occurs concomitantly with the inhibition of glucose uptake. To investigate whether the addition of dmKG indeed led to decreased glucose uptake, we measured extracellular glucose concentration as a function of time during N starvation from cultures of dmKG treated and untreated bacteria. As shown in Fig. 4C, the change in extracellular glucose concentration as a function of time under N starvation did not substantially differ in the media of bacteria treated with dmKG and untreated controls (*P*= 0.7160). However, we note that following the onset of N starvation (N-), the rate of decrease of glucose in the media (i.e. glucose uptake) of untreated bacteria was very low and therefore any differences between the treated and untreated samples would be too small to detect by measuring extracellular glucose concentrations. Hence, we used LC-MS to investigate the intracellular concentration of glucose-6-phosphate (G6P), the metabolic intermediate into which the majority of glucose is converted to during import into the cell. As shown in Fig. 4D, in dmKG untreated bacteria, G6P concentration gradually increased during the first 9 h of N starvation. In contrast, we observed a notable delay in the increase of G6P in the dmKG-treated bacteria. Whilst this difference was not found to be significant at any individual time point, the overall treatment effect across all time points was found to be significant (*P*= 0.0267). We next calculated the rate of change in G6P concentration between time points (calculated as per hour). As shown in Fig. 4E, the rate of change in G6P concentration between N- (when dmKG was added) and N-3 was significantly lower in the dmKG treated cells than in untreated cells. Following N-3, rate of change in G6P concentration largely stabilized between dmKG treated and untreated cells, with rate of change in G6P concentration falling in the untreated cultures by N-6. We note that rate of change in G6P concentration represents the cumulative effect of changes in both uptake and metabolic use. Thus, the results must be interpreted with this consideration in mind. Nonetheless, the results clearly indicate an overall decrease in glucose uptake following dmKG treatment that concomitantly leads to Hfq condensation. We further note that Hfq condensation in the untreated (at N-6) and dmKG treated (at N-3) correlates with a period of decreased change in the concentration of G6P, and thus inhibition of glucose uptake during N starvation. To determine whether Hfq condensation occurs when *E. coli* are N-starved in the presence of a carbon source that does not depend on the PTS system, we repeated the experiments in the presence of lactose as the sole C source (lactose was chosen as it allows a comparable rate of growth as glucose under N-replete conditions [33]). As expected, Hfq-condensates were absent in N- bacteria grown on lactose (Fig. 4F and Supplementary Fig. 2). Hfq condensates were then observed to form as N starvation persisted, with clear condensates observed at N-24. Interestingly, slightly more condensation was observed at N-3 than in cultures with glucose as the C-source. Most notably however, when dmKG was added at N- we observed a marked increase in the number of Hfq condensates as compared to the untreated cultures. Overall, this data suggest that N starvation leads to the inhibition of the PTS system by a process that is associated with Hfq condensation, regardless of the specific carbon source available.

### A role for Hfq condensates in maintaining sRNAs levels during N starvation in *E. coli*

**Figure 5.**
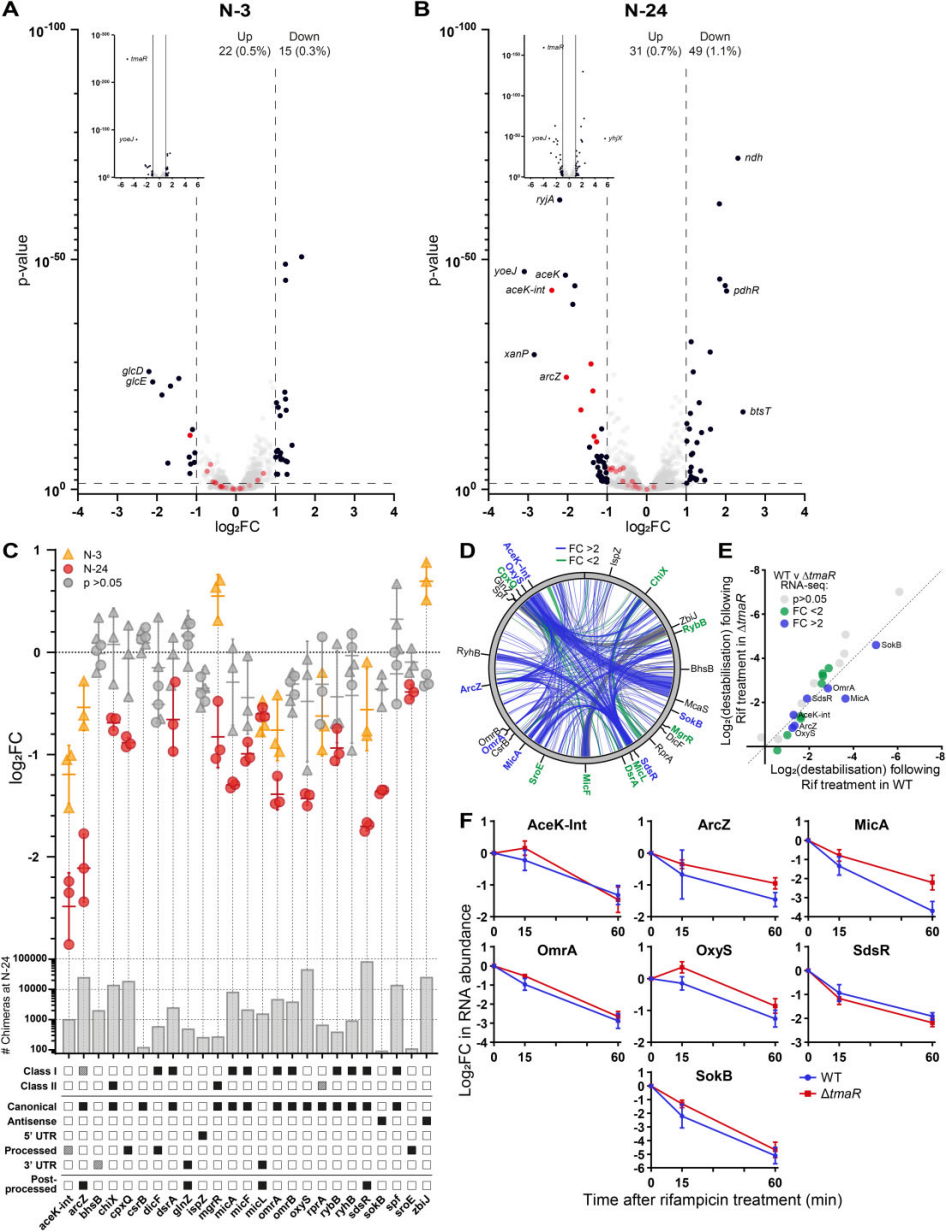
Hfq-condensates contribute to maintaining sRNA levels during N starvation in *E. coli*. (**A**) Volcano plot of differential RNA abundance in the transcriptome of Δ*tmaR E. coli* at N-3 as a log_2_ change from WT bacteria. Analysis performed by DESeq2. RNAs differentially expressed >1 log_2_ (i.e. a greater than two-fold change) are shown in black. RNA differentially expressed >2 log_2_ (i.e. a greater than four-fold change) are labelled. Hfq-sRNA^N-24^ are shown in red. Inset was added to allow viewing of genes with very low *P*-values. The number and percentage (of total detected) of differentially expressed RNAs are indicated at the top right. (**B**) As in panel (A) but for transcriptomes at N-24. (**C**) Dot plot showing log_2_ fold change of individual Hfq-sRNA^N-24^ in Δ*tmaR* bacteria relative to WT bacteria, at N-3 (yellow) and N-24 (red). The sRNAs that were not found to be differentially expressed with adjusted *P*-value > 0.05 by DESeq2 at each timepoint are shown in grey. Lower graph indicates the total number of chimeras found to contain the indicated sRNA at N-24 (across three replicates), as previously determined by RIL-seq [10]. The sRNA class (where experimentally established), the nature of their biogenesis, and whether the sRNA undergoes processing following biogenesis is shown below the dot plot. Dashed squares indicate cases where there is uncertainty whether the given sRNA displays the property listed. (**D**) Circos plot showing RNA–RNA pairs bound to the surface of Hfq in N-24 WT bacteria as previously determined by RIL-seq [10]. sRNAs that are significantly downregulated (*P*< 0.05, FC > 2) in Δ*tmaR E. coli* at N-24 [as in panel (B)] are shown in blue, and those which are partially downregulated (*P*< 0.05, FC < 2) are shown in green. (**E**) Graph showing the log_2_ destabilization of Hfq-sRNA^N-24^ in WT and Δ*tmaR* bacteria, 60 min following treatment with 100 μg/ml of rifampicin at N-24. sRNAs that are significantly downregulated (*P*< 0.05, FC > 2) in Δ*tmaR E. coli* at N-24 [as in panel (B)] are shown in blue, and those which are partially downregulated (*P*< 0.05, FC < 2) are shown in green. sRNAs differentially expressed with FC > 2 are labelled. (**F**) Graphs showing the log_2_ change in RNA abundance of select sRNA, in WT and Δ*tmaR* bacteria, 15- & 60-min following treatment with 100 μg/ml of rifampicin at N-24. RNA abundance is normalized to that at 0-min. sRNAs shown are those determined to be significantly downregulated (*P*< 0.05, FC > 2) in Δ*tmaR E. coli* at N-24, [as shown in panel (B)].

We recently defined the Hfq-mediated RNA–RNA interactions during N starvation [10] and further demonstrated that the absence of Hfq specifically leads to the destabilization of most Hfq-associated sRNA species in N-24 *E. coli* [34]. Given that ∼50% of Hfq molecules are within the Hfq-condensates at N-24, we considered whether the Hfq-condensates have a role in sRNA stabilization. As the Hfq-condensates do not form in Δ*tmaR* bacteria and therefore, unlike dmKG treatment, represent a binary response with respect to Hfq condensation, (i.e. Hfq-condensates are present in WT bacteria and entirely absent in Δ*tmaR* bacteria), we compared the transcriptomes of wild-type and Δ*tmaR* bacteria to determine how the absence of TmaR, *ipso facto*, Hfq-condensates, affects sRNA turnover between N-3 (a time point during N-starvation just prior to detectable Hfq condensation) and N-24 (a time point during N-starvation when Hfq-condensates are fully formed). We defined differentially expressed genes as those with expression levels changed ≥2-fold with a false discovery rate adjusted *P* < 0.05. According to this threshold, the absence of TmaR led to a dysregulation of ∼37 genes (∼22 and ∼15 genes up- and downregulated, respectively), corresponding to ∼0.8% of all genes in *E. coli*, at N-3 (Fig. 5A) and 80 genes (31 and 49 genes up- and downregulated, respectively), corresponding to ∼1.8% of all genes in *E. coli*, at N-24 (Fig. 5B). At N-3, we note that the levels of most sRNAs (25 of 26) that are associated with Hfq at N-24, hereafter referred to as Hfq-sRNA^N-24^, are comparable between WT and Δ*tmaR* bacteria (Fig. 5C). However, at N-24, although there is no detectable difference in Hfq levels in N-24 WT and Δ*tmaR* bacteria (Supplementary Fig. 6), the levels 7 of the 26 (27%) Hfq-sRNA^N-24^ were significantly reduced in Δ*tmaR* bacteria compared WT bacteria (Fig. 5C), with a further 8 being partially downregulated (fold-change < 2). We interpret these findings to suggest that Hfq condensates play a role in stabilizing sRNAs during N starvation. In further support of this, of the 63 sRNAs and ncRNAs which *did not* interact with Hfq at N-24 [10], only 7 (11%) were downregulated in N-24 Δ*tmaR* bacteria (Supplementary Fig. 7). Strikingly, the Hfq-sRNA^N-24^ that were downregulated in N-24 Δ*tmaR* bacteria contribute to ∼64% of previously detected RNA–RNA pairs bound to Hfq at N-24 (Fig. 5D) [10], supporting the notion that the formation of Hfq-condensates facilitates Hfq's role as an sRNA stabilizer during N starvation. We considered whether the Hfq-condensates, once formed, contributed in any way to sRNA stabilization. To investigate this, we obtained the transcriptomes of N-24 WT and Δ*tmaR* bacteria 15- and 60-min following treatment with the transcription inhibitor rifampicin to inhibit *de novo* RNA synthesis (RIF-seq). RNA abundance of all RNA species was normalized to 6S RNA (SsrS), which is known to remain both highly abundant and stable following rifampicin treatment during stationary phase [35]. In Fig. 5E we compare the log_2_-fold destabilization of Hfq-sRNA^N-24^ in WT and Δ*tmaR* bacteria following 60-min treatment with rifampicin. Hfq-sRNA^N-24^ which were found to be significantly downregulated in Δ*tmaR* bacteria at N-24, as in Fig. 5B (*P*< 0.05, FC > 2: AceK-Int, ArcZ, MicA, OmrA, OxyS, SdsR, and SokB) are highlighted in blue, and those partially downregulated (*P*< 0.05, FC < 2: ChiX, CpxQ, DsrA, MgrR, MicF, MicL, RybB, and SroE) are highlighted in green. As further shown in Fig. 5F and Supplementary Fig. 8, the absence of Hfq-condensates did not significantly adversely affect the rate of decay of AceK-Int, ArcZ, ChiX, DsrA, MgrR, MicA, OmrA, OxyS, RybB, SdsR, SokB or SroE, suggesting that although the condensation process of Hfq *per se* contributes to sRNA maintenance during N starvation, once Hfq-condensates have fully formed, they have no detectable influence on sRNA stability in N-24 bacteria. However, this interpretation must be considered with the caveat that sRNA turnover in N-24 bacteria could be sufficiently low to be detectable within the timeframes we conducted the RIF-seq experiments.

**Figure 6.**
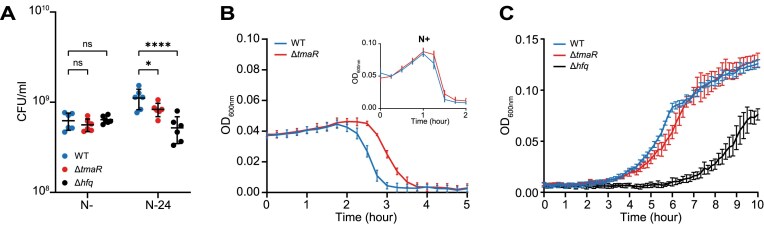
A role of Hfq-condensates in maintaining cellular homeostasis. (**A**) Graph showing proportion of viable cells in the populations of Δ*tmaR* and Δ*hfq* bacteria following 20 min (N-) and 24 h (N-24) of N starvation. Error bars represent standard deviation (*n* = 6). Statistical analysis performed by two-way ANOVA with Dunnett's multiple comparisons test. (**B**) Graph showing collapse of wild-type and Δ*tmaR* N-24 cultures following infection with T7 phage. Insert graph shows collapse of wild-type and Δ*tmaR* N- cultures following infection with T7 phage. Error bars represent standard deviation (*n* = 3). (*P< 0.05; ****P< 0.0001). (**C**) Growth-recovery of WT, Δ*tmaR* and Δ*hfq* N-24 bacteria following subculturing into fresh culture media. Error bars represent standard deviation (*n* = 3).

Although the presence of Hfq condensates did not markedly affect sRNA stability at N-24, we nonetheless considered whether the absence of Hfq-condensates would perturb the RNA–RNA interaction network *ipso facto* post-transcriptional regulation at N-24, which, consequently, could compromise cellular homeostasis. In support of this view, the number of viable cells in the population of N-24 Δ*tmaR* bacteria was ∼25% less than in the N-24 WT population (Fig. 6A). We note that this decrease in viable cells was not as great as that seen in the population of N-24 Δ*hfq* (∼53% decrease) bacteria (Fig. 6A). The difference in viability between N-24 Δ*tmaR* and Δ*hfq* bacteria is perhaps unsurprising since the Hfq molecules not in the condensate in the Δ*tmaR* cells are still able to contribute to adaptive post-transcriptional regulation during N starvation. The efficacy by which bacteriophages infect and replicate in bacteria can serve as an indicator of bacterial metabolic ‘health’. Therefore, we measured how quickly the prototypical *E. coli* bacteriophage T7 replicated and caused collapse of the N-24 WT (Hfq-condensates present) and Δ*tmaR* (Hfq condensates absent) cultures. As shown in Fig. 6B, following addition of T7, the culture of N-24 Δ*tmaR* bacteria collapsed ∼0.5 h after that of the WT bacteria. Notably, this lag in culture collapse was not seen with N+ bacteria, where Hfq-condensates are absent in both the Δ*tmaR* and WT bacteria, confirming a role for Hfq-condensates in maintaining cellular homeostasis. However, whereas N-24 Δ*hfq* bacteria were compromised for growth-recovery from N starvation following inoculation into fresh growth media, Δ*tmaR* bacteria recovered like WT bacteria (Fig. 6C), underscoring a role for Hfq-condensates specifically in the cellular homeostasis during N starvation induced growth-arrest, but not growth-recovery *per se*. Overall, we conclude that the Hfq-condensates contribute to maintenance of sRNA levels during N starvation and thereby to cellular adaptation to N starvation.

## Discussion

Biomolecular condensates formed by RNA and proteins associated with RNA metabolism, ribonucleoprotein condensates, are an emergent and prevalent feature in the subcellular landscape of diverse stressed bacteria (reviewed in [36]). Like analogous condensates such as stress granules or P-bodies in eukaryotic cells [37, 38], bacterial ribonucleoprotein condensates contribute to the adaptive metabolism and homeostasis of RNA. In bacteria, key proteins involved in RNA synthesis, RNA degradation, and RNA regulation have been described to undergo phase condensation. For example, condensates of *E. coli* RNA polymerase have been proposed to serve as hub to store non-transcribing RNA polymerase molecules during growth transitions [39]. The *Caulobacter crescentus* RNase E undergoes condensation and forms a multiprotein complex called the BR-body that confers *C. crescentus* cells stress resistance [40, 41]. Although the primary function of BR-bodies is to facilitate RNA decay [40], the complex network of protein-protein interactions that underpin their formation suggests that BR-bodies could have multiple roles in RNA metabolism in *C. crescentus* [42]. Further, RNases in several other bacteria, such as RNase Y in *Bacillus subtilis* [43] and RNase J in *Helicobacter pylori* [44] form condensates, contributing to RNA decay. Recently, the *E. coli* RNA chaperone CsrA was described to form condensates with the RNA-degradation machinery and regulate the expression of virulence genes [45].

The most ubiquitous bacterial RNA chaperone Hfq, the subject of this study, forms polar condensates in response to diverse stresses in *E. coli* (see Introduction). Despite the prevalence of ribonucleoprotein condensates in stressed bacteria, most studies to date have focused on understanding their function in stress physiology through determining their composition. We have now shown that Hfq condensation is an adaptive response to N starvation that occurs independently of, but alongside, the reprogramming of gene expression during N starvation. Inspired by the observation made by the Amster-Choder laboratory revealing that Hfq condensates co-localize with condensates of a novel glucose uptake regulator TmaR [13], our study has now revealed that the formation of Hfq-condensates occurs concomitantly with inhibition of glucose uptake in N-starved *E. coli*. An important breakthrough in our study is the finding that Hfq-condensates could be induced by αKG – the key metabolite involved in coordinating nitrogen and carbon metabolism in *E. coli*. It is well established that intracellular concentration of αKG increases upon entry into N starvation but does not rise further during long-term N starvation [46] as it is found in the supernatants of N-starved cultures (suggesting active export of αKG to prevent its hyperaccumulation inside cells) [47]. Thus, we propose that Hfq condensation is unlikely to be due to the accumulation of αKG as a function of time during N starvation. Notably, isothermal titration calorimetry and dynamic light scattering assays showed that αKG does not bind to Hfq (our unpublished data). Therefore, the effect of αKG on Hfq is likely to be indirect, and independent of TmaR, as TmaR-condensates were present prior to the onset of N starvation and were not detectably affected by αKG. Nonetheless, our results show that Hfq condensation can be induced by αKG, which simultaneously inhibits glucose uptake via the PTS enzyme E1. Additionally, the dependency of Hfq-condensates on TmaR (another inhibitor of glucose uptake through the same enzyme) highlights the necessity for the functional interaction between Hfq- and TmaR-condensates to effectively suppress glucose uptake in N-starved bacteria. The observation that Hfq-condensates do not disperse when N-24 bacteria are resuspended into fresh media devoid of N but containing glucose (Fig. 2) is further consistent with this view. Further still, the fact that both the ordinary and dmKG-inducible formation of Hfq condensates occurs when bacteria are N-starved in non-PTS carbon sources (e.g. lactose) supports the need to inhibit the PTS-system upon N-starvation, regardless of carbon source in the environment. Whilst this interpretation may seem counterintuitive, it is not unreasonable to consider that, in response to excess carbon, N-starved bacteria would supress glucose uptake even if no glucose were present. Our work has clearly uncovered a link between the regulation of glucose-uptake and Hfq condensation, however, we cannot exclude that other factors such as redox stress (which can cause RNA damage), αKG-mediated changes to cAMP-signalling, modulation of TCA cycle activity and overall perturbed N metabolism etc. could also precipitate Hfq condensation (see later).

The overwhelming majority of bacterial cells contain Hfq-condensates following 24 h under N starvation. Therefore, we regard Hfq-condensates that exist in N-24 bacteria as fully formed. By leveraging the finding that Hfq-condensates do not form in N-24 Δ*tmaR* bacteria and our previous data describing the transcriptome [34] and genome-wide Hfq-associated RNA–RNA interaction network [10] at N-24, we have now revealed a function for Hfq-condensates in maintaining sRNA levels during N starvation in *E. coli*. However, we note that we were unable to detect any difference in sRNA stability at N-24, i.e. once Hfq-condensates are fully formed. In the context of our experimental timeframe, i.e. 24 h of N starvation, this result could mean that Hfq-condensates contribute to sRNA stability during early stages of N starvation, i.e. prior to reaching the N-24 state, suggesting that the adaptive response to N starvation occurs in a temporally phasic manner. We note that a limitation of the conclusion that Hfq-condensates contribute to sRNA stabilization during N starvation is the absence of evidence showing that the downregulated sRNA species in N-24 Δ*tmaR* bacteria are indeed physically associated with the Hfq-condensates, but this will be subject of future studies. Nonetheless, our conclusions are consistent with and expand results of a recent study from the Jakob laboratory showing Hfq-condensates that form under N starvation function to preserve polyadenylated mRNAs (which would otherwise be degraded) and mRNA encoding components of the translation machinery [17]. We previously reported that condensates of the RNA degradosome colocalize with Hfq-condensates in N-starved *E. coli* [11]. Therefore, in light of our new results, we propose that condensates of Hfq and the RNA degradosome potentially interact to coordinate the selective preservation and degradation of RNA molecules during N starvation.

The bacterial transcriptional response to N-starvation is well-understood and is primarily triggered by the NtrBC system in *E. coli* and related bacteria [4]. At the post-transcriptional level, the Hfq-mediated RNA–RNA interaction network undergoes large-scale changes upon entry into and during N starvation, underscoring the importance of the adaptive post-transcriptional response to N starvation [10]. Pertinent to this study, the abundance of the Hfq-dependent sRNA GlnZ increases upon entry into N starvation [9]. One of the targets of GlnZ is *sucA*, which encodes a subunit of αKG-dehydrogenase, the enzyme which catalyses the conversion of αKG to succinyl-CoA in the Krebs cycle. GlnZ downregulates the expression of *sucA*. In light of our new results, one could consider the possibility that GlnZ redirects αKG from the Krebs cycle to trigger Hfq condensation and concomitantly the inhibition of glucose uptake and stabilization of Hfq-associated sRNAs. However, this is unlikely to be case as the efficacy by which ordinary and dmKG-induced Hfq-condensation occurs in Δ*glnZ* bacteria does not detectably differ from that in WT bacteria (Supplementary Fig. 9). We further note that GlnZ levels do not differ between N-24 WT and Δ*tmaR* bacteria (Fig. 5C). Interestingly, GlnZ also targets *tmaR* but the regulatory consequence of this interaction on *tmaR* expression is presently unclear. GlnZ has also been shown to repress *aceE* mRNA by enabling its degradation in an Hfq-dependent manner [9]. Additionally, we note that the mRNA upregulated in N-24 Δ*tmaR* bacteria, i.e. when Hfq-condensates are absent, include *aceE* (E1 component of pyruvate dehydrogenase (PDH) complex) *aceF* (E2 component of the PDH complex) and *pdhR* (the transcriptional regulator of the PDH complex). In *E. coli*, the PDH complex catalyses the conversion of pyruvate to acetyl-CoA, a crucial step linking glycolysis to the Krebs cycle. The finding that these genes are upregulated in the absence of Hfq-condensates, i.e. N-24 Δ*tmaR* bacteria further underscores the association of Hfq-condensates in the regulation of carbon flux through the Krebs cycle.

In sum, our study has shown that Hfq-condensates are associated with the inhibition of glucose uptake in N-starved *E. coli* and suggests that Hfq-condensation is required for coordinating carbon and N metabolism under certain stresses. Additionally, our study has expanded the current perception in the field that Hfq-condensates contribute to just preserving mRNA molecules [17]. Although both of these functions of Hfq-condensates might not be mutually inclusive, Hfq-condensates clearly contribute to maintaining cellular homeostasis during N starvation. Most bacterial stress ribonucleoprotein condensates, including Hfq-condensates, are heterotypic in nature and their formation is underpinned by multivalent interactions between different proteins and different types of RNA molecules. Although we are now beginning to understand why they form and how they contribute to stress adaption, their heterotypic nature implies that neither their formation is triggered by a universal ‘stress signal’ nor they are restricted in their functions. Instead, as bacteria must respond and adapt to and survive temporally changing conditions, it is likely that the formation of ribonucleoprotein condensates and their composition is precipitated by a combination of diverse cellular and physical factors and contribute to different adaptive, including RNA metabolism associated, functions in the cell.

## Supplementary Material

gkaf1006_Supplemental_Files

## Data Availability

The RNA-seq discussed in this publication is accessible through ArrayExpress: E-MTAB-15469.
