# Supplementary material for: Condensation of the RNA chaperone Hfq is coupled to inhibition of glucose uptake and contributes to the stabilization of regulatory RNAs in nitrogen-starved Escherichia coli
Source: Nucleic Acids Res. 2025 Oct 21;53(19):gkaf1006. doi: 10.1093/nar/gkaf1006 (PMC12539622; doi:10.1093/nar/gkaf1006)
Supplement: gkaf1006_Supplemental_Files [file gkaf1006_supplemental_files.zip › Supplementary Figures_.docx]

**
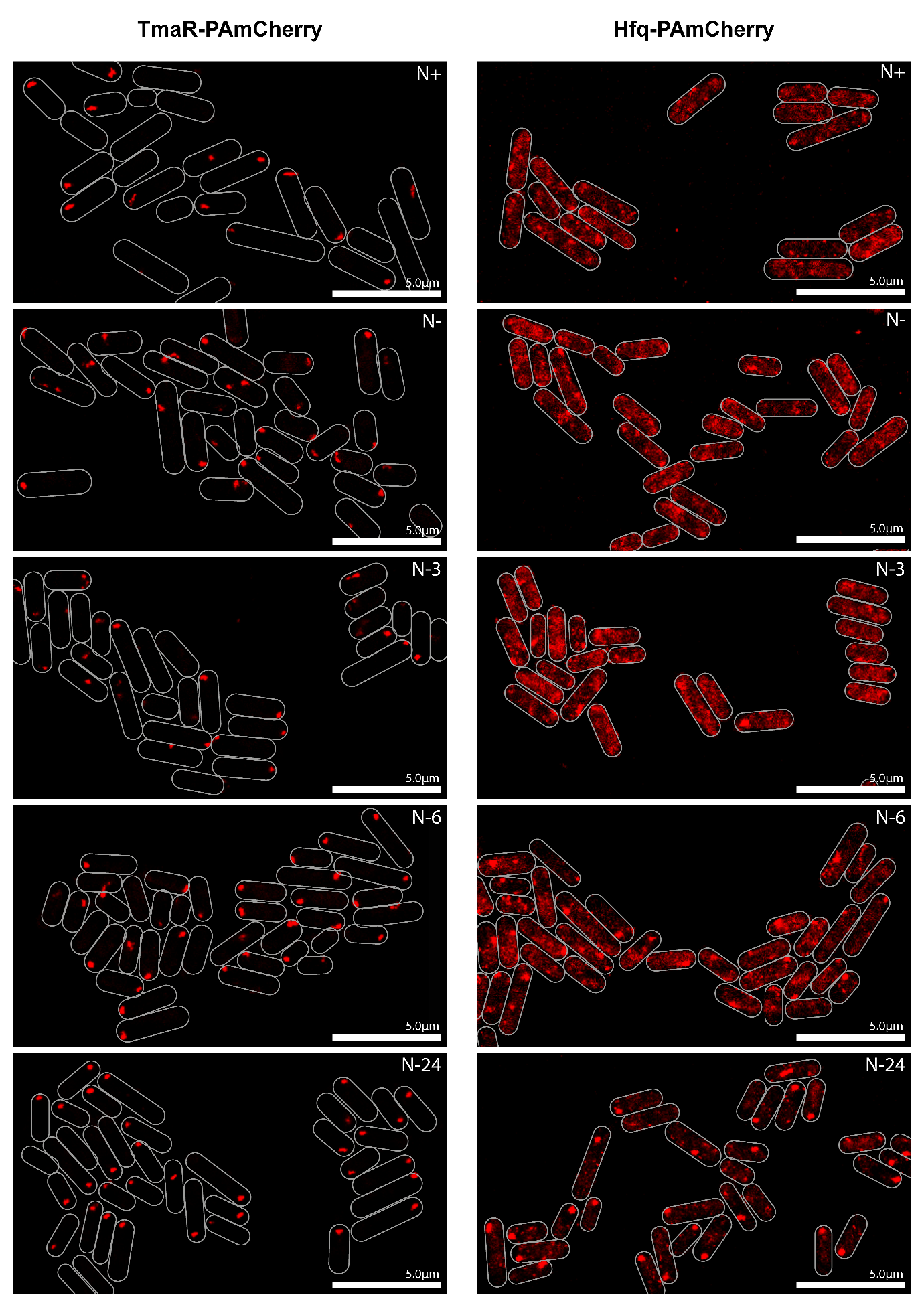
**

**Supplementary Figure 1.**

Larger representative PALM images of TmaR (left) and Hfq (right) in *E. coli* as a function of time under N starvation. Images were taken at the indicated time points. These are examples of the data used in **Figure 3B** and are included to provide a more comprehensive view of condensate formation dynamics during N starvation.

**
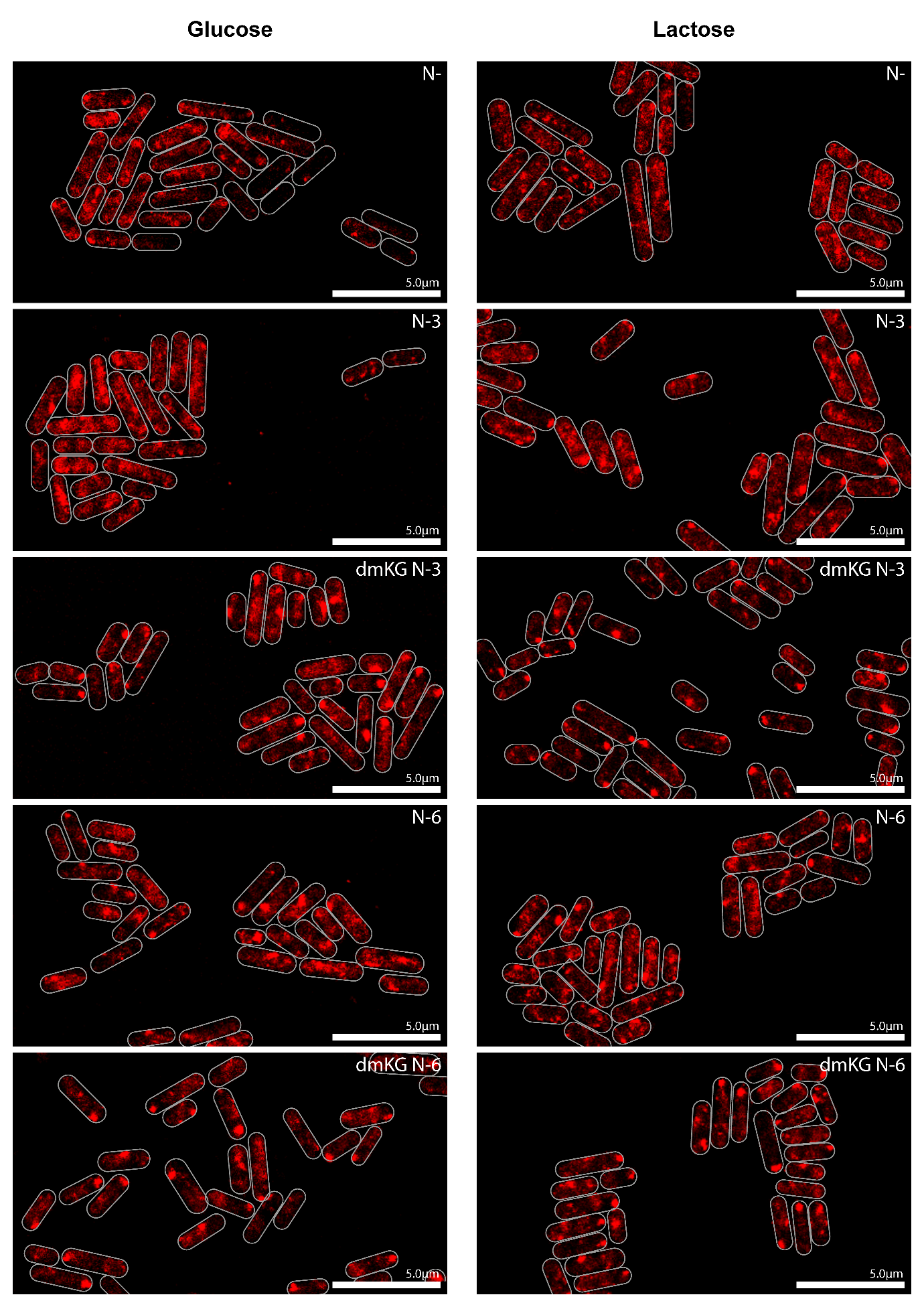
**

**Supplementary Figure 2.**

Larger representative PALM images of Hfq in *E. coli* as a function of time under N starvation, with and without treatment with 40mM dmKG at N-. Bacteria were grown with either glucose (left) or lactose (right) as the sole carbon source. Images were taken at the indicated time points. These images are included to provide a more comprehensive view of dmKG-dependant induction of Hfq condensates.

**
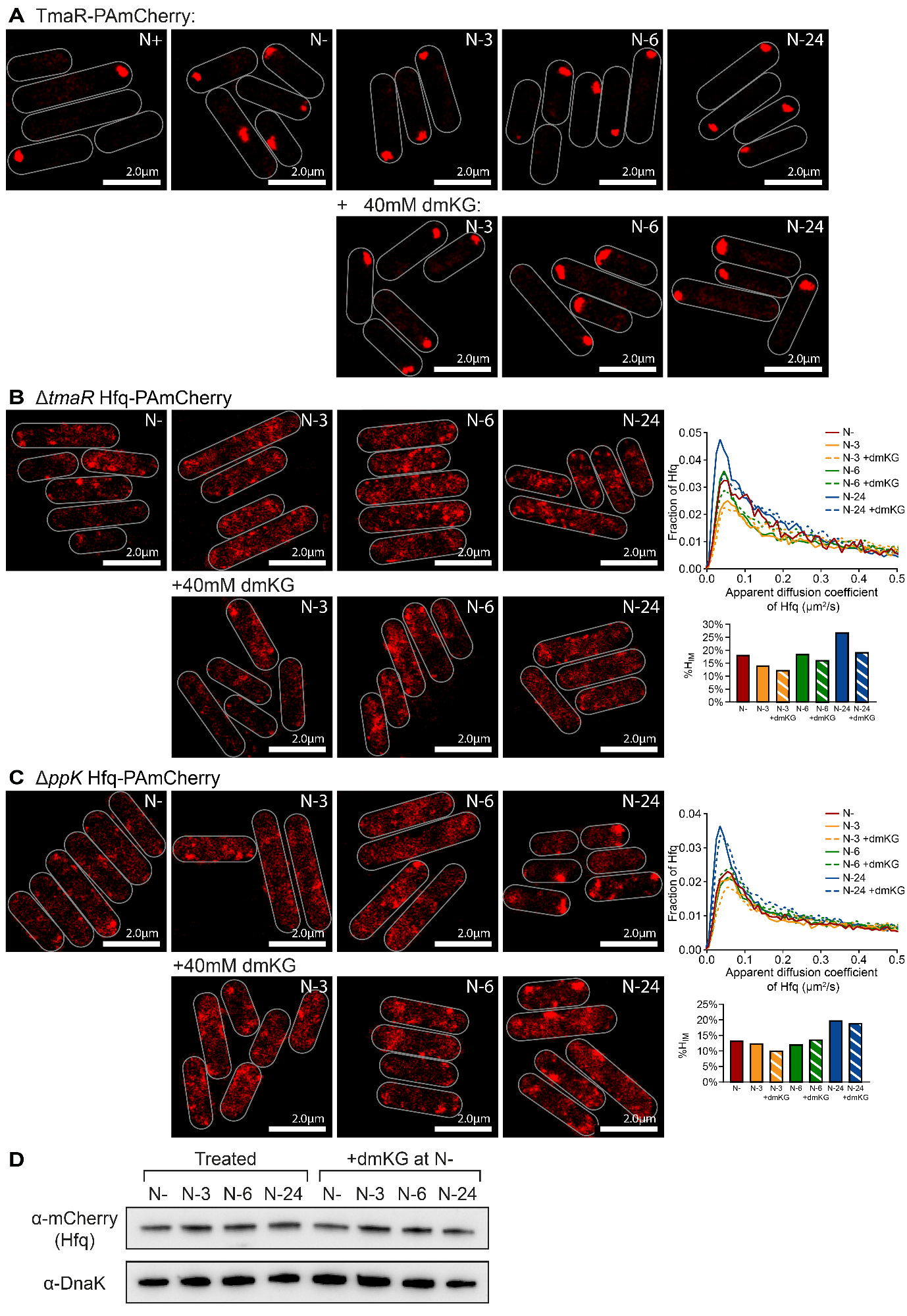
**

**Supplementary Figure 3.**

**(A)** Representative PALM images of TmaR in *E. coli* experiencing N starvation with and without treatment with 40mM dmKG at N-. The untreated images are the same as those used in Figure 3A and are only included for ease of comparison. **(B)** As in (A) but of Hfq in Δ*tmaR* bacteria. **(C)** As in B but in Δ*ppK* bacteria. In **(B)** and **(C)**, the graphs show the distribution of apparent diffusion coefficient of Hfq molecules at the different sampling time points and the corresponding %H_IM_. **(D)** Representative immunoblot of whole-cell extracts of wild-type *E. coli* containing Hfq translationally fused to PAmCherry, sampled at N-, N-3, N-6 N-24, with and without treatment with 40mM dmKG at N-. Probed with anti-mCherry antibody (for Hfq) and anti-DnaK antibody (loading control).

**
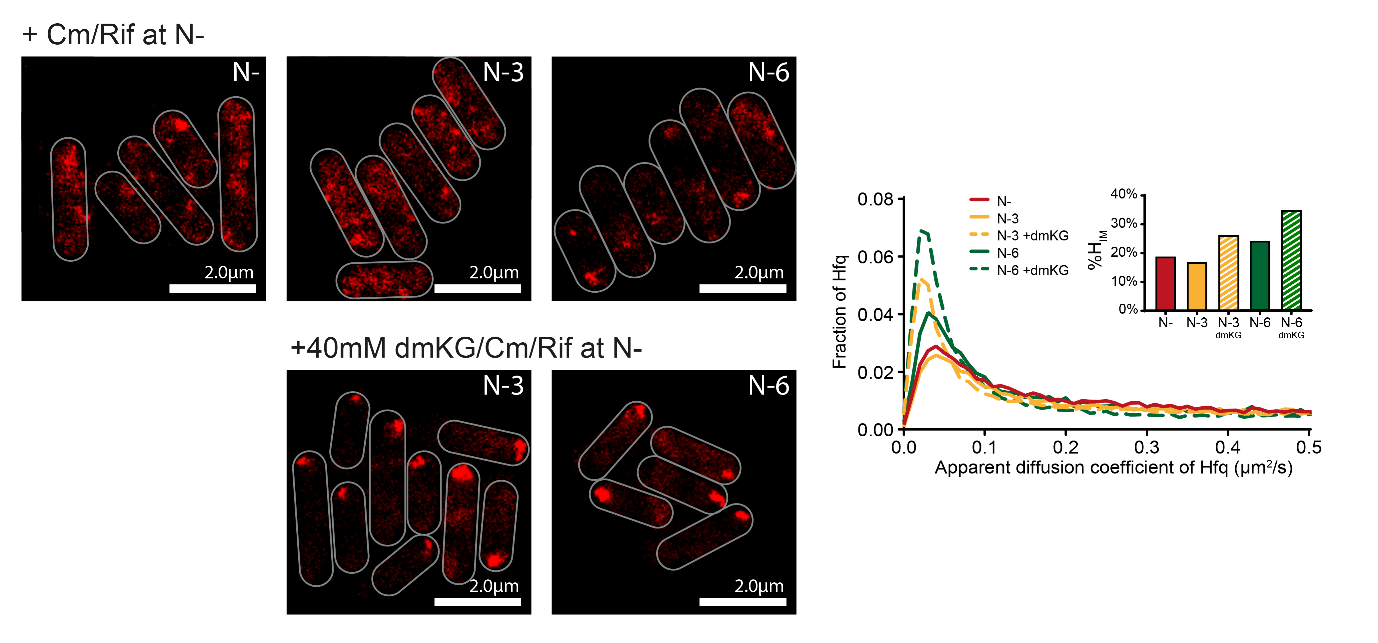
**

**Supplementary Figure 4.**

Representative PALM images of Hfq in *E. coli* experiencing N starvation, treated with 100μg/ml rifampicin and 150μg/ml chloramphenicol at N-, with and without treatment with 40mM dimethyl-ketoglutarate (dmKG) at N-. Graphs show the distribution of apparent diffusion coefficient of Hfq molecules at the different sampling time points and the corresponding %H_IM_.

**
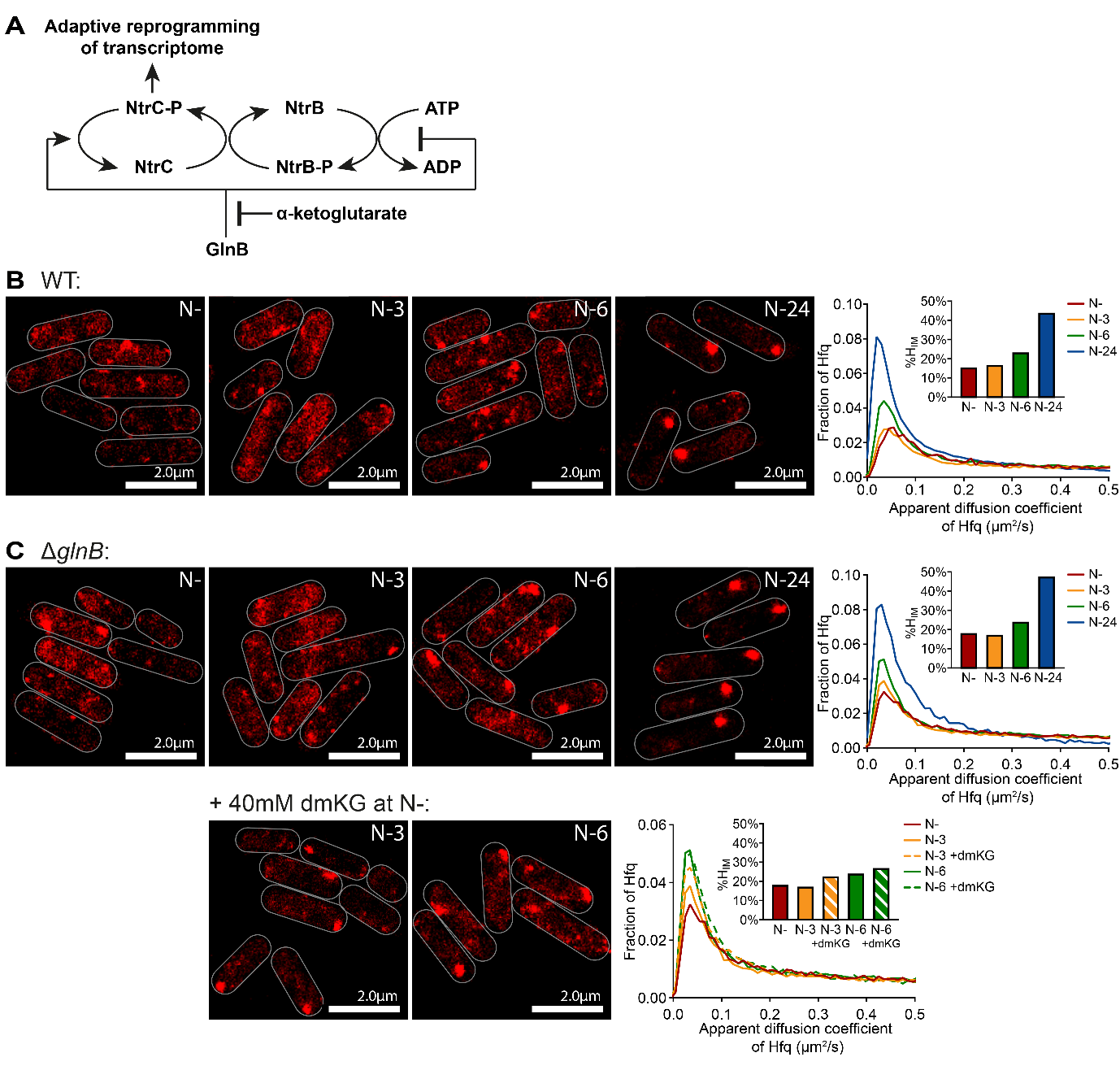
**

**Supplementary Figure 5.**

**(A)** Schematic showing GlnB and αKG mediated regulation of the NtrBC two-component system during nitrogen starvation. **(B)** Representative PALM images of Hfq in *E. coli* experiencing N starvation. Graphs showing the distribution of apparent diffusion coefficient of Hfq molecules at the different sampling time points and the corresponding %H_IM_. **(C)** as in **(B)** but for Δ*glnB* bacteria, with and without treatment with 40mM dimethyl-ketoglutarate (dmKG) at N-.

**
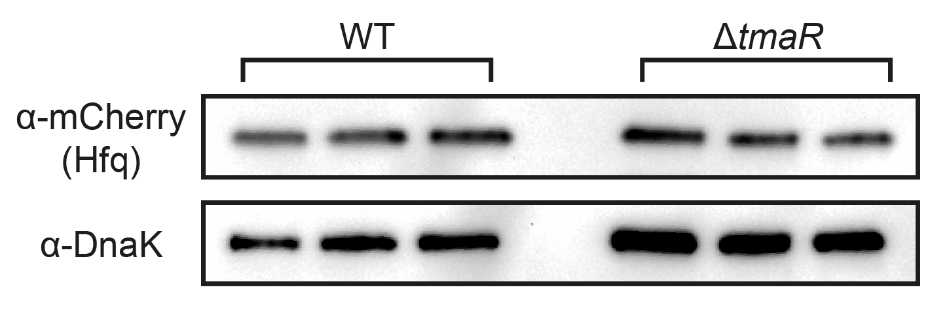
**

**Supplementary Figure 6.**

Immunoblot of whole-cell extracts of wild-type and Δ*tmaR* *E. coli* containing Hfq translationally fused to PAmCherry, sampled at N-24, three biological replicates are shown. Probed with anti-mCherry antibody (for Hfq) and anti-DnaK antibody (loading control).

**
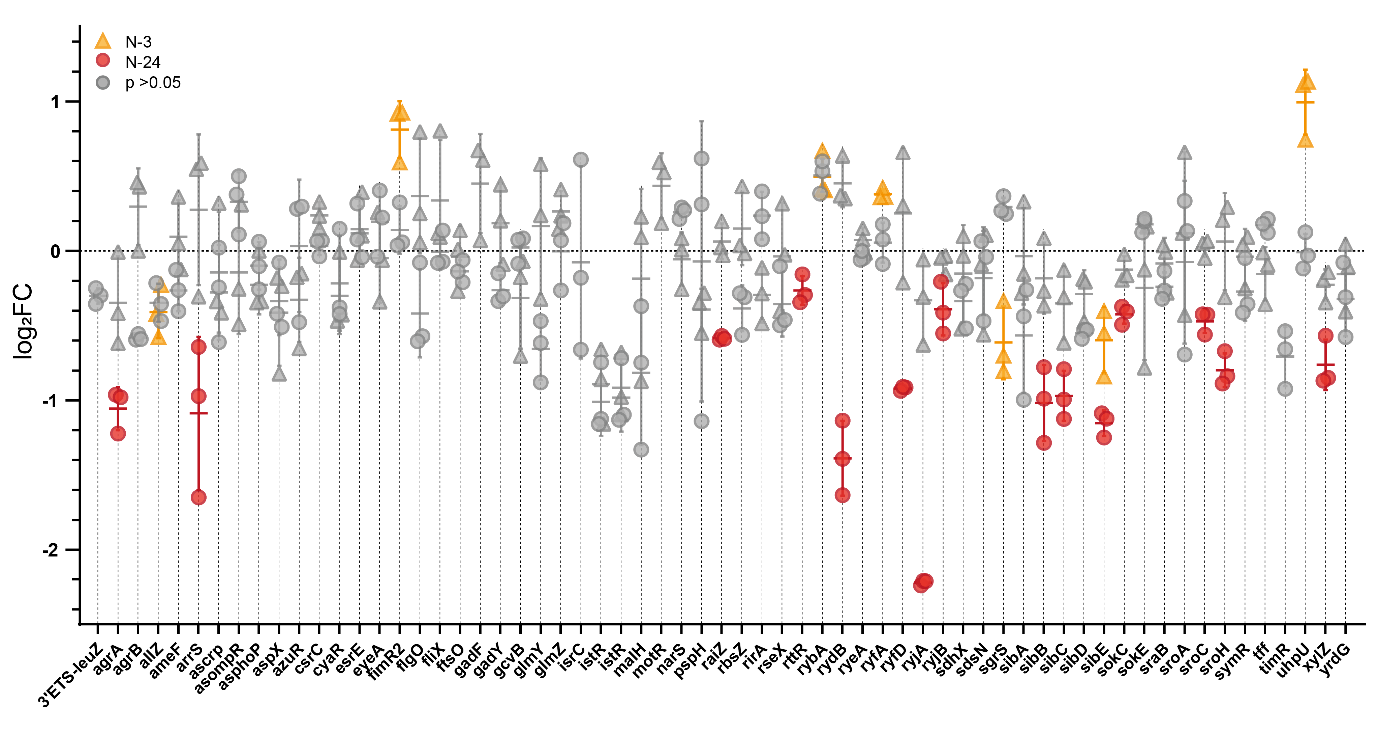
**

**Supplementary Figure 7.**

Dot plot showing log_2_ fold change of individual sRNA and non-coding RNA previously shown not to interact with Hfq at N-24 in Δ*tmaR* bacteria relative to WT bacteria, at N-3 (Yellow) and N-24 (Red). The RNAs that were not found to be differentially expressed with adjusted p-value >0.05 by DESeq2 at each timepoint are shown in grey. RNA with mapped to less than 10 reads during RNA-seq were excluded.

**
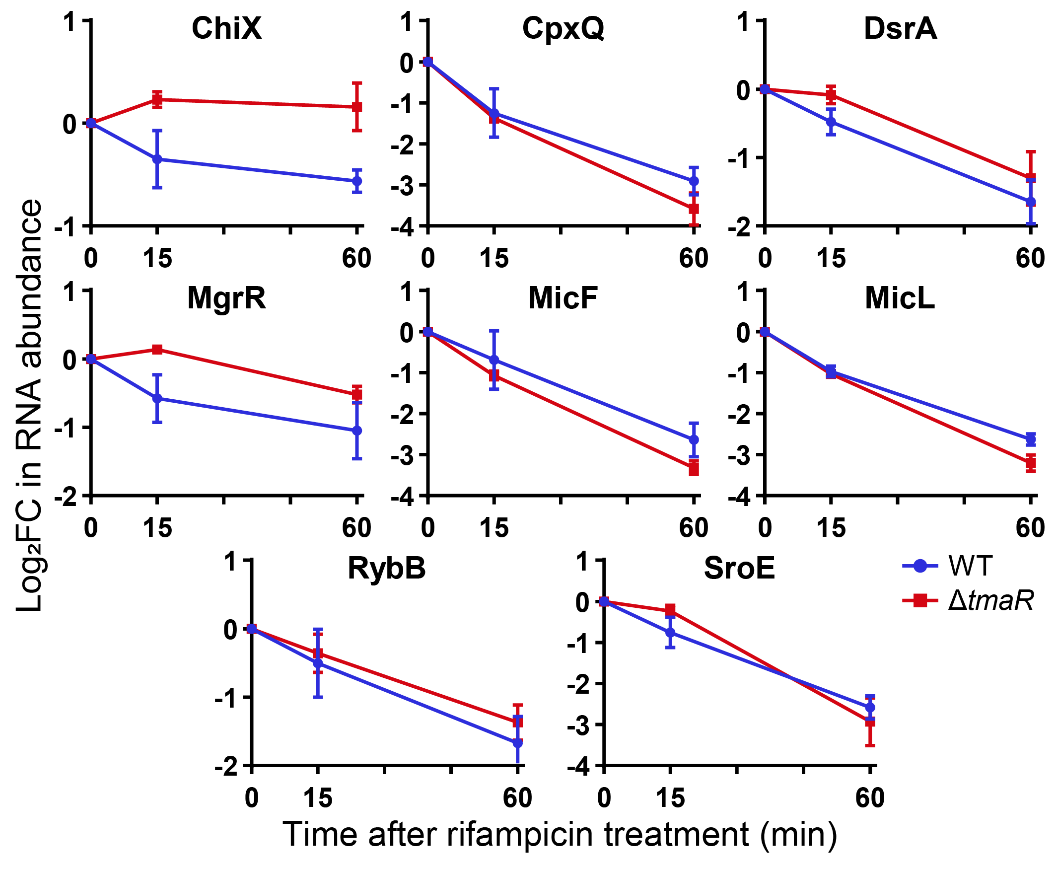
**

**Supplementary Figure 8.**

Graphs showing the log_2_ change in RNA abundance of select sRNA, in in WT and Δ*tmaR* bacteria, 15- & 60-min following treatment with 100μg/ml of rifampicin at N-24. RNA abundance is normalised to that at 0-min. sRNA shown are those determined to be partially downregulated (p<0.05, FC<2) in Δ*tmaR* bacteria at N-24, (as shown in Figure 5B)

**
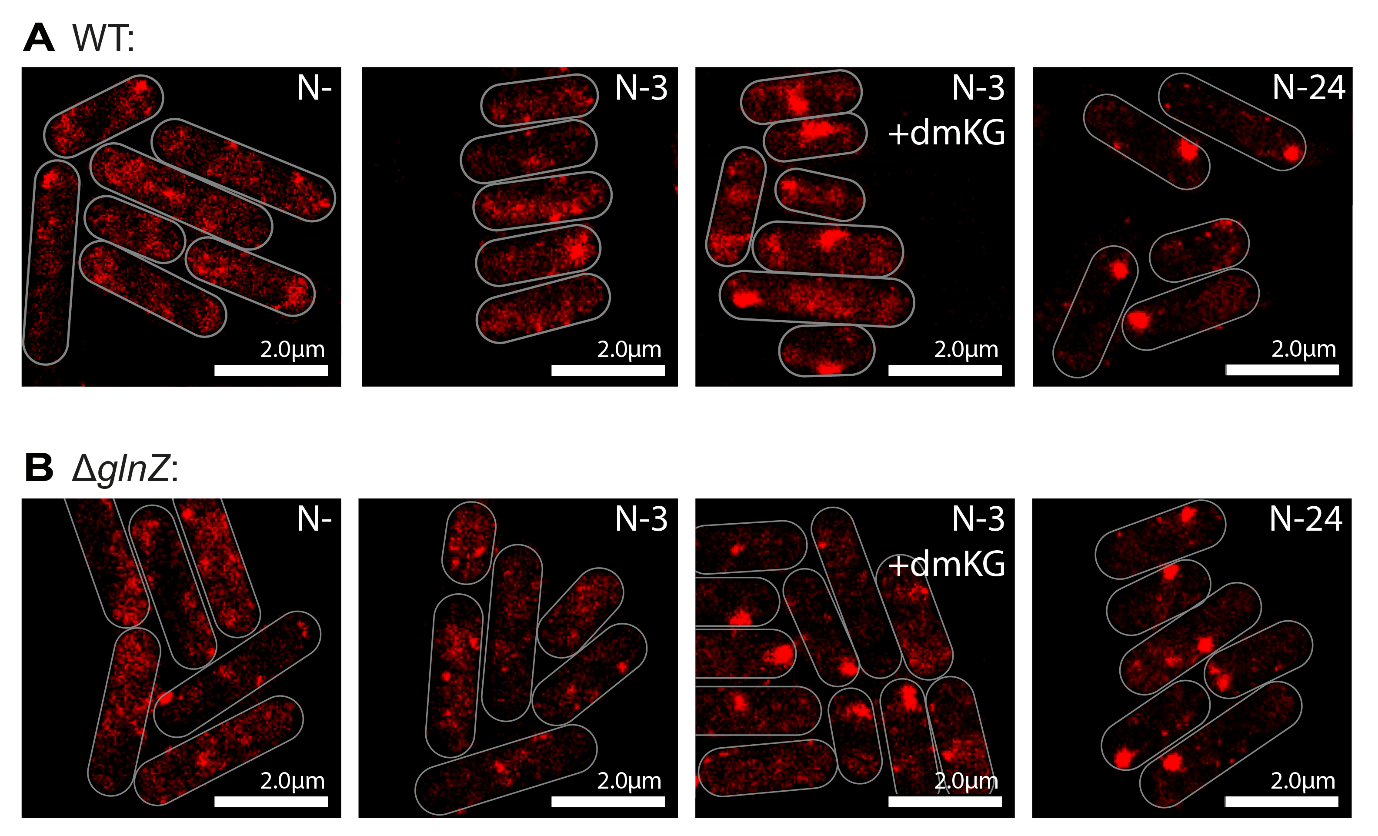
**

**Supplementary Figure 9.**

**(A)** Representative PALM images of Hfq in *E. coli* experiencing N starvation with and without treatment with 40mM dimethyl-ketoglutarate (dmKG) at N-. The first three images are the same as those used in Figure 3A and are only included for ease of comparison. **(B)** as in **(A)** but for Δ*glnZ* bacteria.

**Supplementary Table 1.**

| *E. coli* strains used in this study: | |  |  |
| --- | --- | --- | --- |
| Name | Description | | Source or reference |
| Wild-type (MG1655) | *E. coli* K-12 *rph-*1 | | *E. coli* Genetic Stock Center |
| Hfq-PAmCherry | MG1655 *hfq-PAmCherry-kan* | | [1] |
| TmaR-PAmCherry | MG1655 *tmaR-PAmCherry-kan* | | This Study |
| Δ*tmaR* (BW25113) | BW25113 Δ*tmaR::kan* | | [2] |
| Δ*ppK* (BW25113) | BW25113 Δ*ppK::kan* | | [2] |
| Δ*glnB* (BW25113) | BW25113 Δ*glnB::kan* | | [2] |
| Δ*glnZ* (MG1655) | MG1655 Δ*glnZ::kan* | | [3] – Provided by Storz lab, NIH |
| Δ*tmaR* Hfq-PAmCherry | Hfq-PAmCherry Δ*tmaR::kan* | | This Study |
| Δ*ppK* Hfq-PAmCherry | Hfq-PAmCherry Δ*ppK::kan* | | This Study |
| Δ*glnB* Hfq-PAmCherry | Hfq-PAmCherry Δ*glnB::kan* | | This Study |
| Δ*glnZ* Hfq-PAmCherry | Hfq-PAmCherry Δ*glnZ::kan* | | This Study |
| Δ*tmaR* (MG1655) | MG1655 Δ*tmaR::kan* | | This Study |
| Δ*hfq* (MG1655) | MG1655 Δ*hfq::kan* | | [1] |

1. McQuail, J., Switzer, A., Burchell, L. and Wigneshweraraj, S. (2020) The RNA-binding protein Hfq assembles into foci-like structures in nitrogen starved Escherichia coli. The Journal of biological chemistry, 295, 12355-12367.
2. Baba, T., Ara, T., Hasegawa, M., Takai, Y., Okumura, Y., Baba, M., Datsenko, K.A., Tomita, M., Wanner, B.L. and Mori, H. (2006) Construction of Escherichia coli K-12 in-frame, single-gene knockout mutants: the Keio collection. *Mol Syst Biol*, **2**, 2006 0008.
3. Walling, L.R., Kouse, A.B., Shabalina, S.A., Zhang, H. and Storz, G. (2022) A 3' UTR-derived small RNA connecting nitrogen and carbon metabolism in enteric bacteria. *Nucleic Acids Res*, **50**, 10093-10109.
